# Supplementary material for: Strong sesquiterpene emissions from Amazonian soils
Source: Nat Commun. 2018 Jun 8;9:2226. doi: 10.1038/s41467-018-04658-y (PMC5993744; doi:10.1038/s41467-018-04658-y)
Supplement: Supplementary file 1 — Supplementary information [file 41467_2018_4658_MOESM1_ESM.pdf]

nature COMMUNICATIONS

Supplementary information for

Strong sesquiterpene emissions from Amazonian soils

Bourtsoukidis et, al.

Sample number	Depth	Ecosyst.	Pit	Coordinates	Elev. (m)	Soil type	BD (g/cm^3)	pH	Clay (%)	Σ_{RB}(m mol kg^{-1})	Sum of bases (cmol kg^{-1})	Index Π	T (^oC)	Conditions
1	0 - 5 cm												30	Aerobic
2													30	Aerobic
3		Terra Firme (TF1)	Pit 1	02 08'06" S, 59 00'07"W	127	Ferralsol	0,51	3,6	81	7,79	2,45	0	30	Aerobic
4													30	Aerobic
5													30	Anaerobic
6													20	Aerobic
7		Terra Firme (TF2)	Pit 2	02 08'13" S, 58 59'31"W	145	Ferralsol	0,53	3,5	81	7,8	3,41	0	30	Aerobic
8													30	Anaerobic
9													30	Aerobic
10		Terra Firme (TF3)	Pit 3 (ATTO)	2 08'38.8"S, 58 59'59.6"W	126	Ferralsol	0,52	n.d.	n.d.	n.d.	n.d.	n.d.	20	Aerobic
11													30	Anaerobic
12													20	Flood
13		Terra Firme (TF4)	Pit 4	13 04'35.53"S, 52 22'36.61"W	246	Ferralsol	0,86*	4,6	48,9*	28,3*	2,32*	0*	30	Aerobic
14													30	Aerobic
15		Terra Firme (TF5)	Pit 5	13 04'44.73"S, 52 23'10.21"W	378	Ferralsol	0,86*	4,5	48,9*	28,3*	2,32*	0*	30	Aerobic
16													30	Aerobic
17													20	Aerobic
18		Floodplain Terrace (FLT1)	Pit 6	02 13'24" S, 59 01'56"W	40	Alisol	0,83	3,7	65	20,59	2,38	2	30	Aerobic
19													30	Anaerobic
20													20	Aerobic
21		Floodplain Terrace (FLT2)	Pit 7	02 14'03.5" S, 59 01'58,4"W	30	Alisol	0,87	3,5	64,5	22,57	4,18	1	30	Aerobic
22													30	Anaerobic
23													20	Aerobic
24		White sand (WS)	Pit 8	2 11'24.9"S, 59 01'13"W	36	Podzol / Arenosol	n.d.	n.d.	n.d.	n.d.	n.d.	n.d.	20	Aerobic
25													20	Aerobic
26	10 - 15 cm	Terra Firme (TF1)	Pit 1	02 08'06" S, 59 00'07"W	127	Ferralsol	0,81	4,2	85	n.d.	0,51	n.d.	30	Anaerobic
27													20	Aerobic
28		Terra Firme (TF2)	Pit 2	02 08'13" S, 58 59'31"W	145	Ferralsol	0,80	3,8	83	n.d.	0,99	n.d.	30	Anaerobic
29													20	Aerobic
30		Terra Firme (TF3)	Pit 3 (ATTO)	2 08'38.8"S, 58 59'59.6"W	126	Ferralsol	0,80	n.d.	n.d.	n.d.	n.d.	n.d.	30	Anaerobic
31													20	Aerobic
32		Floodplain Terrace (FLT1)	Pit 6	2 13'24"S, 59 01'56"W	40	Alisol	1,18	3,8	65	n.d.	0,71	n.d.	30	Anaerobic
33													30	Aerobic
34		Floodplain Terrace (FLT2)	Pit 7	02 14'03,5" S, 59 01'58,4"W	30	Alisol	1,11	4,3	62	n.d.	1,24	n.d.	30	Anaerobic
35		White sand (WS)	Pit 8	2 11'24.9"S, 59 01'13"W	36	Podzol / Arenosol	n.d	n.d.	n.d.	n.d.	n.d.	n.d.	20	Aerobic
36	45 - 50 cm	Terra Firme (TF1)	Pit 1	02 08'06" S, 59 00'07"W	127	Ferralsol	1,09	4,5	81	n.d.	0,25	n.d.	20	Aerobic
37		Terra Firme (TF2)	Pit 2	02 08'13" S, 58 59'31"W	145	Ferralsol	1,07	4,3	86,5	n.d.	0,45	n.d.	20	Aerobic
38		Terra Firme (TF3)	Pit 3 (ATTO)	2 08'38.8"S, 58 59'59.6"W	126	Ferralsol	n.d.	n.d.	n.d.	n.d.	n.d.	n.d.	20	Aerobic
39													20	Aerobic

40	Floodplain Terrace (FLT1)	Pit 6	02 13'24" S, 59 01'56"W	40	Alisol	1,29	4,3	65,5	n.d.	0,42	n.d.	20	Aerobic
41	Floodplain Terrace (FLT2)	Pit 7	2 14'03.5"S, 59 01'58.4"W	30	Alisol	1,19	4,5	71	n.d.	0,72	n.d.	20	Aerobic
42	White sand (WS)	Pit 8	2 11'24.9"S, 59 01'13"W	36	Podzol / Arenosol	n.d.	n.d.	n.d.	n.d.	n.d.	n.d.	20	Aerobic

Supplementary Table 1 │ Sample characteristics and treatments. An overview of the soil dessication experiments is presented for the different soil types, depths, temperature and conditions. All plateau soils were classified as Ferralsol with advanced state of pedogenetic development, low nutrient and high clay content. Terrace soils were classified as Alisols, which are intermediate aged soils with relatively high element content on a clay bases. Similarly, total reserve bases (Σ_{RB}; the sum of total cations Ca, Mg, K and Na are presented only for the first 10cm of soil depth) was approximately three times higher in the terrace soils indicating that these soils display a lower pedogenetic development compared to the TF soils. Anaerobic conditions were achieved by the use of cleaned compressed air and anaerobic by using a pressurized bottle of nitrogen (6.0). As “flood” we refer to a single experiment (S9) where anaerobic conditions were achieved by flooding the soil sample to 3 times its soil saturation capacity. Incubation temperature indicates the temperature inside each chamber. The values that were not defined are indicated with n.d. while the * symbol indicates the values for a pit in the vicinity (12°55’15.49’’ S , 52°22’22.75’’W).

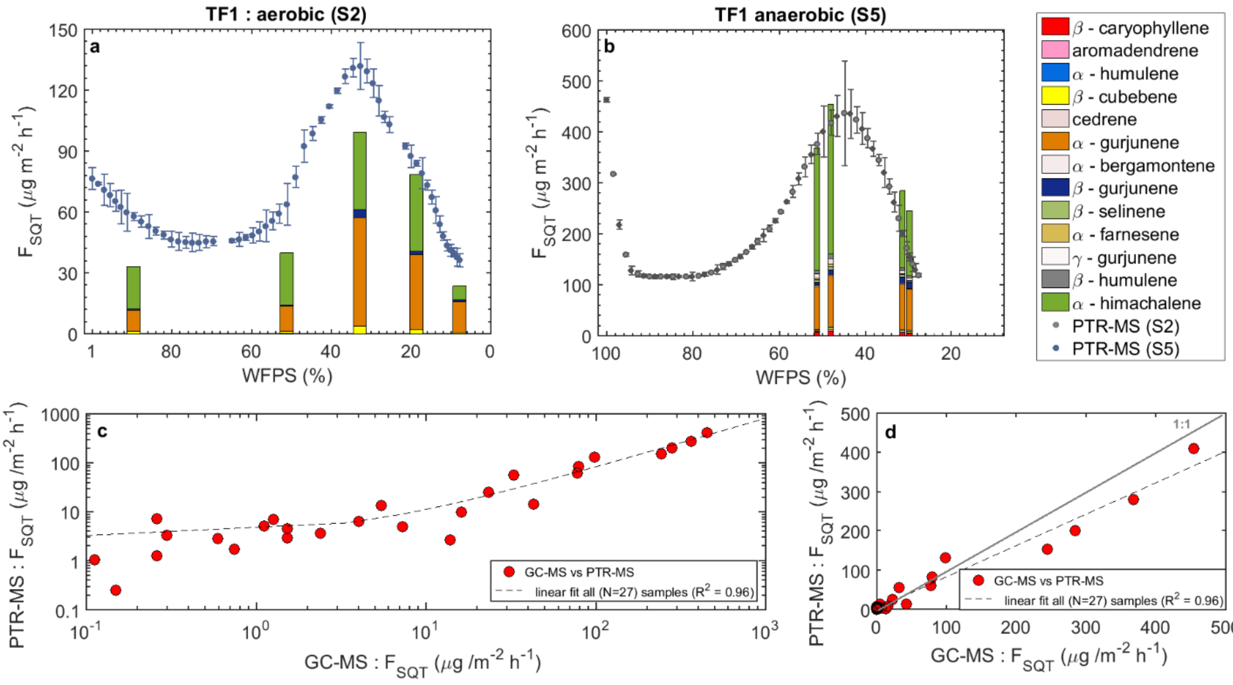

Supplementary Figure 1 │ Comparison of PTR-MS and GC-MS measurements. During a. aerobic (sample S2) and b. anaerobic (sample S5) desiccation of TF1 soils. The markers indicate the measurement points from PTR-MS and the error bars the std (N=4) of each measurement point. The stacked bar plots indicate the GC-MS measurements with the respective SQT species to be denoted at the colored legend on the right. In c, all simultaneous measurements (N = 27) between PTR-MS and GC-MS appear as red circles together with a linear regression fit. In d, the same points are plotted in linear scale to illustrate the possible underestimation of the PTR-MS measurements.
